# Supplementary material for: Assessment of Immunogenic and Antigenic Properties of Recombinant Nucleocapsid Proteins of Five SARS-CoV-2 Variants in a Mouse Model
Source: Viruses. 2023 Jan 13;15(1):230. doi: 10.3390/v15010230 (PMC9861333; doi:10.3390/v15010230)
Supplement: Supplementary file 1 [file viruses-15-00230-s001.zip › viruses-2174436-supplementary.pdf]

Supplementary Materials

Table S1. Amino acid substitutions between the N protein used for immunization and the N proteins of different SARS-CoV-2 strains used as antigens in ELISA.

Immunogen	Antigen				
	B.1 (Wuhan)	B.1.351 (Beta)	P.1 (Gamma)	B.1.617.2 (Delta)	B.1.1.529 (Omicron)
B.1 (Wuhan)	-	P13S, T205I	P80R, R203K, G204R	D63G, R203M, G215C, D377Y, R385K	P13L, ERS31-33del, R203K, G204R
B.1.351 (Beta)	S13P, I205T	-	S13P, P80R, R203K, G204R, I205T	S13P, D63G, R203M, I205T, G215C, D377Y, R385K	S13L, ERS31-33del, R203K, G204R, I205T
P.1 (Gamma)	R80P, K203R, R204G	P13S, R80P, K203R, R204G, T205I	-	D63G, R80P, K203M, R204G, G215C, D377Y, R385K	P13L, ERS31-33del, R80P
B.1.617.2 (Delta)	G63D, M203R, C215G, Y377D, K385R	P13S, G63D, M203R, T205I, C215G, Y377D, K385R	G63D, P80R, M203K, G204R, C215G, Y377D, K385R	-	P13L, ERS31-33del, G63D, M203K, G204R, C215G, Y377D, K385R
B.1.1.529 (Omicron)	L13P, ERS31-33ins, K203R, R204G	L13S, ERS31-33ins, K203R, R204G, T205I	L13P, ERS31-33ins, P80R	L13P, ERS31-33ins, D63G, K203M, R204G, G215C, D377Y, R385K	-

Table S2. B-cell epitopes of SARS-CoV-2 N protein of B.1 (Wuhan) strain deposited in the Immune Epitope Database which contain variable amino acid residues.

Position	IEDB ID	Epitope sequence	Mutation	Strain	Method	Reference
1-15	1313173	MSDNGPQNQRNAPRI	P13S, P13L	B.1.351, B.1.1.529	microarray	[1,2]
1-16	1680361	MSDNGPQNQRNAPRIT	P13S, P13L	B.1.351, B.1.1.529	microarray	[3]
1-20	1392311	MSDNGPQNQRNAPRITFGGP	P13S, P13L	B.1.351, B.1.1.529	ELISA	[4]
1-39	1392312	MSDNGPQNQRNAPRITFGGPS DSTGSNQNGERSGARSKQ	P13S, P13L, 31-33ERS del	B.1.351, B.1.1.529	phage display	[5]
2-17	1692539	SDNGPQNQRNAPRITF	P13S, P13L	B.1.351, B.1.1.529	microarray	[3]

3-18	1651733	DNGPQNQRNAPRITFG	P13S, P13L	B.1.351, B.1.1.529	microarray	[3]
4-18	1541724	NGPQNQRNAPRITFG	P13S, P13L	B.1.351, B.1.1.529	microarray	[1]
4-19	1681860	NGPQNQRNAPRITFGG	P13S, P13L	B.1.351, B.1.1.529	microarray	[3]
5-16	1443077	GPQNQRNAPRIT	P13S, P13L	B.1.351, B.1.1.529	microarray	[6]
5-20	1662374	GPQNQRNAPRITFGGP	P13S, P13L	B.1.351, B.1.1.529	microarray	[3]
6-17	1489379	PQNQRNAPRITF	P13S, P13L	B.1.351, B.1.1.529	microarray	[6]
6-20	1323019	PQNQRNAPRITFGGP	P13S, P13L	B.1.351, B.1.1.529	ELISA, microarray	[2,7]
6-21	1686370	PQNQRNAPRITFGGPS	P13S, P13L	B.1.351, B.1.1.529	microarray	[3]
7-18	1494133	QNQRNAPRITFG	P13S, P13L	B.1.351, B.1.1.529	microarray	[6]
7-21	2001265	QNQRNAPRITFGGPS	P13S, P13L	B.1.351, B.1.1.529	microarray	[1]
7-22	1387278	QNQRNAPRITFGGPSD	P13S, P13L	B.1.351, B.1.1.529	microarray	[3]
7-27	1387279	QNQRNAPRITFGGPSDSTGSN	P13S, P13L	B.1.351, B.1.1.529	microarray	[3]
8-22	1392332	NQRNAPRITFGGPSD	P13S, P13L	B.1.351, B.1.1.529	ELISA	[7]
9-20	1494864	QRNAPRITFGGP	P13S, P13L	B.1.351, B.1.1.529	microarray	[6]
9-23	1313380	QRNAPRITFGGPSDS	P13S, P13L	B.1.351, B.1.1.529	microarray	[8]
9-24	1387377	QRNAPRITFGGPSDST	P13S, P13L	B.1.351, B.1.1.529	microarray	[3]
10-24	1542300	RNAPRITFGGPSDST	P13S, P13L	B.1.351, B.1.1.529	microarray	[1]
10-25	1387793	RNAPRITFGGPSDSTG	P13S, P13L	B.1.351, B.1.1.529	microarray	[3]
11-26	1385708	NAPRITFGGPSDSTGS	P13S, P13L	B.1.351, B.1.1.529	microarray	[3]
11-30	1392317	NAPRITFGGPSDSTGSNQNG	P13S, P13L	B.1.351, B.1.1.529	ELISA	[4]
12-27	1377774	APRITFGGPSDSTGSN	P13S, P13L	B.1.351, B.1.1.529	microarray	[3]
13-23	1343959	PRITFGGPSDS	P13S, P13L	B.1.351, B.1.1.529	microarray	[8]
13-27	1313309	PRITFGGPSDSTGSN	P13S, P13L	B.1.351, B.1.1.529	microarray	[1,8]
13-28	1686445	PRITFGGPSDSTGSNQ	P13S, P13L	B.1.351, B.1.1.529	microarray	[3]
16-31	1697782	TFGGPSDSTGSNQNGE	31E del	B.1.1.529	microarray	[3]
17-32	1656956	FGGPSDSTGSNQNGER	31-32ER del	B.1.1.529	microarray	[3]
18-33	1661033	GGPSDSTGSNQNGERS	31-33ERS del	B.1.1.529	microarray	[3]
18-39	1892918	GGPSDSTGSNQNGERSGARSKQ	31-33ERS del	B.1.1.529	ELISA	[9]

19-33	2001081	GPSDSTGSNQNGERS	31-33ERS del	B.1.1.529	microarray	[1]
19-34	1662386	GPSDSTGSNQNGERSG	31-33ERS del	B.1.1.529	microarray	[3]
20-35	1686525	PSDSTGSNQNGERSGA	31-33ERS del	B.1.1.529	microarray	[3]
21-36	1692591	SDSTGSNQNGERSGAR	31-33ERS del	B.1.1.529	microarray	[3]
21-40	1392416	SDSTGSNQNGERSGARSKQR	31-33ERS del	B.1.1.529	ELISA	[4]
21-59	1392417	SDSTGSNQNGERSGARSKQRR PQGLPNNTASWFTALTQH	31-33ERS del	B.1.1.529	phage display	[5]
22-36	1539968	DSTGSNQNGERSGAR	31-33ERS del	B.1.1.529	microarray	[1]
22-37	1652355	DSTGSNQNGERSGARS	31-33ERS del	B.1.1.529	microarray	[3]
23-38	1695939	STGSNQNGERSGARSK	31-33ERS del	B.1.1.529	microarray	[3]
24-35	1514563	TGSNQNGERSGA	31-33ERS del	B.1.1.529	microarray	[6]
24-38	1542758	TGSNQNGERSGARSK	31-33ERS del	B.1.1.529	ELISA	[7]
24-39	1698184	TGSNQNGERSGARSKQ	31-33ERS del	B.1.1.529	microarray	[3]
24-44	1870816	TGSNQNGERSGARSKQRRPQG	31-33ERS del	B.1.1.529	ELISA	[10]
25-36	1444073	GSNQNGERSGAR	31-33ERS del	B.1.1.529	microarray	[6]
25-39	1312611	GSNQNGERSGARSKQ	31-33ERS del	B.1.1.529	microarray	[1]
25-40	1662825	GSNQNGERSGARSKQR	31-33ERS del	B.1.1.529	microarray	[3]
26-40	1542535	SNQNGERSGARSKQR	31-33ERS del	B.1.1.529	ELISA	[7]
26-41	1694848	SNQNGERSGARSKQRR	31-33ERS del	B.1.1.529	microarray	[3]
27-42	1683330	NQNGERSGARSKQRRP	31-33ERS del	B.1.1.529	microarray	[3]
28-42	1542153	QNGERSGARSKQRRP	31-33ERS del	B.1.1.529	ELISA, microarray	[1,7]
28-43	1387259	QNGERSGARSKQRRPQ	31-33ERS del	B.1.1.529	microarray	[3]
28-55	1387260	QNGERSGARSKQRRPQGLPNN TASWFTA	31-33ERS del	B.1.1.529	microarray	
29-44	1385895	NGERSGARSKQRRPQG	31-33ERS del	B.1.1.529	microarray	[3]
30-41	1439579	GERSGARSKQRR	31-33ERS del	B.1.1.529	microarray	[6]
30-45	1381027	GERSGARSKQRRPQGL	31-33ERS del	B.1.1.529	microarray	[3]
31-45	1397276	ERSGARSKQRRPQGL	31-33ERS del	B.1.1.529	microarray	[1,2]
31-46	1379994	ERSGARSKQRRPQGLP	31-33ERS del	B.1.1.529	microarray	[3]
31-50	1392143	ERSGARSKQRRPQGLPNNTA	31-33ERS del	B.1.1.529	ELISA	[4]
32-47	1387915	RSGARSKQRRPQGLPN	32-33RS del	B.1.1.529	microarray	[3]
33-44	1503974	SGARSKQRRPQG	33S del	B.1.1.529	microarray	[6]
33-47	1313553	SGARSKQRRPQGLPN	33S del	B.1.1.529	microarray	[8]
33-48	1388305	SGARSKQRRPQGLPNN	33S del	B.1.1.529	microarray	[3]
44-63	2060826	GLPNNTASWFTALTQHGKED	D63G	B.1.617.2	ELISA	[11]
49-63	1313733	TASWFTALTQHGKED	D63G	B.1.617.2	microarray	[1]
50-64	1310292	ASWFTALTQHGKEDL	D63G	B.1.617.2	ELISA	[7]
51-66	1696659	SWFTALTQHGKEDLKF	D63G	B.1.617.2	microarray	[3]
51-70	1392442	SWFTALTQHGKEDLKFPRGQ	D63G	B.1.617.2	ELISA	[4]
52-66	1543345	WFTALTQHGKEDLKF	D63G	B.1.617.2	microarray	[1]
54-65	1512359	TALTQHGKEDLK	D63G	B.1.617.2	microarray	[6]
54-68	1512360	TALTQHGKEDLKFPR	D63G	B.1.617.2	microarray	[6]
55-69	2000982	ALTQHGKEDLKFPRG	D63G	B.1.617.2	microarray	[1]
56-70	1321406	LTQHGKEDLKFPRGQ	D63G	B.1.617.2	ELISA	[7]
58-72	1542112	QHGKEDLKFPRGQGV	D63G	B.1.617.2	microarray	[1]
58-73	1687902	QHGKEDLKFPRGQGVP	D63G	B.1.617.2	microarray	[3]
59-73	1334464	HGKEDLKFPRGQGVP	D63G	B.1.617.2	ELISA	[12]
59-74	1664380	HGKEDLKFPRGQGVPI	D63G	B.1.617.2	microarray	[3]
60-75	1661476	GKEDLKFPRGQGVPIN	D63G	B.1.617.2	microarray	[3]
61-76	1669657	KEDLKFPRGQGVPINT	D63G	B.1.617.2	microarray	[3]
61-80	1392232	KEDLKFPRGQGVPINTNSSP	D63G	B.1.617.2	ELISA	[4]
62-73	1426591	EDLKFPRGQGVP	D63G	B.1.617.2	microarray	[6]

62-76	1540035	EDLKFPRGQGVPINT	D63G	B.1.617.2	ELISA	[7]
62-77	1653592	EDLKFPRGQGVPINTN	D63G	B.1.617.2	microarray	[3]
63-78	1651371	DLKFPRGQGVPINTNS	D63G	B.1.617.2	microarray	[3]
65-80	1669963	KFPRGQGVPINTNSSP	P80R	P.1	microarray	[3]
66-80	1316855	FPRGQGVPINTNSSP	P80R	P.1	ELISA	[7]
66-81	1658360	FPRGQGVPINTNSSPD	P80R	P.1	microarray	[3]
67-81	2001245	PRGQGVPINTNSSPD	P80R	P.1	microarray	[1]
67-82	1686429	PRGQGVPINTNSSPDD	P80R	P.1	microarray	[3]
68-82	1542258	RGQGVPINTNSSPDD	P80R	P.1	ELISA	[7]
68-83	1690210	RGQGVPINTNSSPDDQ	P80R	P.1	microarray	[3]
69-80	1443234	GQGVPINTNSSP	P80R	P.1	microarray	[6]
69-84	1662438	GQGVPINTNSSPDDQI	P80R	P.1	microarray	[3]
70-81	1492723	QGVPINTNSSPD	P80R	P.1	microarray	[6]
70-84	1542109	QGVPINTNSSPDDQI	P80R	P.1	microarray	[1]
70-85	1687879	QGVPINTNSSPDDQIG	P80R	P.1	microarray	[3]
71-86	1663542	GVPINTNSSPDDQIGY	P80R	P.1	microarray	[3]
71-90	1392199	GVPINTNSSPDDQIGYYRRA	P80R	P.1	ELISA	[4]
72-83	1527506	VPINTNSSPDDQ	P80R	P.1	microarray	[6]
72-86	1543205	VPINTNSSPDDQIGY	P80R	P.1	ELISA	[7]
72-87	1705162	VPINTNSSPDDQIGYY	P80R	P.1	microarray	[3]
73-84	1487555	PINTNSSPDDQI	P80R	P.1	microarray	[6]
73-87	1313287	PINTNSSPDDQIGYY	P80R	P.1	microarray	[1]
73-88	1685533	PINTNSSPDDQIGYYR	P80R	P.1	microarray	[3]
74-85	1453361	INTNSSPDDQIG	P80R	P.1	microarray	[6]
74-88	1540832	INTNSSPDDQIGYYR	P80R	P.1	ELISA	[7]
74-89	1667534	INTNSSPDDQIGYYRR	P80R	P.1	microarray	[3]
75-86	1484451	NTNSSPDDQIGY	P80R	P.1	microarray	[6]
75-89	1484452	NTNSSPDDQIGYYRR	P80R	P.1	microarray	[6]
75-90	1683919	NTNSSPDDQIGYYRRA	P80R	P.1	microarray	[3]
76-90	1542862	TNSSPDDQIGYYRRA	P80R	P.1	microarray	[1]
77-91	1313250	NSSPDDQIGYYRRAT	P80R	P.1	ELISA	[12]
78-93	1695669	SSPDDQIGYYRRATRR	P80R	P.1	microarray	[3]
79-90	1507694	SPDDQIGYYRRA	P80R	P.1	microarray	[6]
79-93	2001314	SPDDQIGYYRRATRR	P80R	P.1	microarray	[1]
79-94	1694961	SPDDQIGYYRRATRRI	P80R	P.1	microarray	[3]
80-94	1541910	PDDQIGYYRRATRRI	P80R	P.1	ELISA	[7]
188-203	1695383	SRSRNSSRNSTPGSSR	R203M, R203K	P.1, B.1.617.2, B.1.1.529	microarray	[3]
189-204	1691371	RSRNSSRNSTPGSSRG	R203M, R203K, G204R	P.1, B.1.617.2, B.1.1.529	microarray	[3]
190-204	1542569	SRNSSRNSTPGSSRG	R203M, R203K, G204R	P.1, B.1.617.2, B.1.1.529	microarray	[1]
190-205	1695346	SRNSSRNSTPGSSRGT	R203M, R203K, G204R, T205I	B.1.351, P.1, B.1.617.2, B.1.1.529	microarray	[3]
191-205	1397406	RNSSRNSTPGSSRGT	R203M, R203K, G204R, T205I	B.1.351, P.1, B.1.617.2, B.1.1.529	microarray	[2]

191-206	1690946	RNSSRNSTPGSSRGTS	R203M, R203K, G204R, T205I	B.1.351, P.1, B.1.617.2, B.1.1.529	microarray	[3]
191-210	1392403	RNSSRNSTPGSSRGTSPARM	R203M, R203K, G204R, T205I	B.1.351, P.1, B.1.617.2, B.1.1.529	ELISA	[4]
192-207	1683686	NSSRNSTPGSSRGTSP	R203M, R203K, G204R, T205I	B.1.351, P.1, B.1.617.2, B.1.1.529	microarray	[3]
193-207	1313680	SSRNSTPGSSRGTSP	R203M, R203K, G204R, T205I	B.1.351, P.1, B.1.617.2, B.1.1.529	microarray	[1]
193-208	1695708	SSRNSTPGSSRGTSPA	R203M, R203K, G204R, T205I	B.1.351, P.1, B.1.617.2, B.1.1.529	microarray	[3]
194-209	1695349	SRNSTPGSSRGTSPAR	R203M, R203K, G204R, T205I	B.1.351, P.1, B.1.617.2, B.1.1.529	microarray	[3]
195-210	1690951	RNSTPGSSRGTSPARM	R203M, R203K, G204R, T205I	B.1.351, P.1, B.1.617.2, B.1.1.529	microarray	[3]
196-210	1397391	NSTPGSSRGTSPARM	R203M, R203K, G204R, T205I	B.1.351, P.1, B.1.617.2, B.1.1.529	microarray	[1,2]
196-211	1683723	NSTPGSSRGTSPARMA	R203M, R203K, G204R, T205I	B.1.351, P.1, B.1.617.2, B.1.1.529	microarray	[3]
197-212	1696051	STPGSSRGTSPARMAG	R203M, R203K, G204R, T205I	B.1.351, P.1, B.1.617.2, B.1.1.529	microarray	[3]
198-213	1699690	TPGSSRGTSPARMAGN	R203M, R203K, G204R, T205I	B.1.351, P.1, B.1.617.2, B.1.1.529	microarray	[3]
199-213	2001233	PGSSRGTSPARMAGN	R203M, R203K, G204R, T205I	B.1.351, P.1, B.1.617.2, B.1.1.529	microarray	[1]
199-214	1685320	PGSSRGTSPARMAGNG	R203M, R203K, G204R, T205I	B.1.351, P.1, B.1.617.2, B.1.1.529	microarray	[3]

200-215	1662875	GSSRGTSPARMAGNGG	R203M, R203K, G204R, T205I, G215C	B.1.351, P.1, B.1.617.2, B.1.1.529	microarray	[3]
201-216	1695698	SSRGTSPARMAGNGGD	R203M, R203K, G204R, T205I, G215C	B.1.351, P.1, B.1.617.2, B.1.1.529	microarray	[3]
201-220	1392436	SSRGTSPARMAGNGGDAALA	R203M, R203K, G204R, T205I, G215C	B.1.351, P.1, B.1.617.2, B.1.1.529	ELISA	[4]
201-239	1392437	SSRGTSPARMAGNGGDAALAL LLLDRLNQLESKMSGKGQ	R203M, R203K, G204R, T205I, G215C	B.1.351, P.1, B.1.617.2, B.1.1.529	phage display	[5]
202-216	1542565	SRGTSPARMAGNGGD	R203M, R203K, G204R, T205I, G215C	B.1.351, P.1, B.1.617.2, B.1.1.529	microarray	[1]
202-217	1695296	SRGTSPARMAGNGGDA	R203M, R203K, G204R, T205I, G215C	B.1.351, P.1, B.1.617.2, B.1.1.529	microarray	[3]
203-215	1313679	SSRGTSPARMAGNGG	R203M, R203K, G204R, T205I	B.1.351, P.1, B.1.617.2, B.1.1.529	microarray	[1]
203-217	1497839	RGTSPARMAGNGGDA	R203M, R203K, G204R, T205I, G215C	B.1.351, P.1, B.1.617.2, B.1.1.529	microarray	[6]
203-218	1690239	RGTSPARMAGNGGDAA	R203M, R203K, G204R, T205I	B.1.351, P.1, B.1.617.2, B.1.1.529	microarray	[3]
204-219	1663184	GTSPARMAGNGGDAAL	G204R, T205I	P.1, B.1.617.2, B.1.1.529	microarray	[3]
205-219	1313804	TSPARMAGNGGDAAL	G215C	B.1.617.2	microarray	[1]
205-220	1700343	TSPARMAGNGGDAALA	T205I, G215C	B.1.351, B.1.617.2	microarray	[3]
206-220	1542543	SPARMAGNGGDAALA	G215C	B.1.617.2	ELISA	[7]
206-221	1694946	SPARMAGNGGDAALAL	G215C	B.1.617.2	microarray	[3]
208-222	1539579	ARMAGNGGDAALALL	G215C	B.1.617.2	microarray	[1]
208-223	1377826	ARMAGNGGDAALALLL	G215C	B.1.617.2	microarray	[3]
208-238	1377827	ARMAGNGGDAALALLLLDRL NQLESKMSGKG	G215C	B.1.617.2	microarray	[3]
210-224	1541577	MAGNGGDAALALLLL	G215C	B.1.617.2	ELISA	[7]
210-225	1385332	MAGNGGDAALALLLLD	G215C	B.1.617.2	microarray	[3]
210-226	1475537	MAGNGGDAALALLLLDR	G215C	B.1.617.2	microarray	[6]
211-225	1314288	AGNGGDAALALLLLD	G215C	B.1.617.2	microarray	[1]
211-226	1377496	AGNGGDAALALLLLDR	G215C	B.1.617.2	microarray	[3]

211-230	1392036	AGNGGDAALALLLLDRLNQL	G215C	B.1.617.2	ELISA	[4]
212-226	1540534	GNGGDAALALLLLDR	G215C	B.1.617.2	ELISA	[7]
212-227	1381497	GNGGDAALALLLLDRL	G215C	B.1.617.2	microarray	[3]
213-228	1385897	NGGDAALALLLLDRLN	G215C	B.1.617.2	microarray	[3]
214-228	1540456	GGDAALALLLLDRLN	G215C	B.1.617.2	ELISA, microarray	[1,7]
214-229	1381105	GGDAALALLLLDRLNQ	G215C	B.1.617.2	microarray	[3]
215-230	1074903	GDAALALLLLDRLNQL	G215C	B.1.617.2	microarray	[3]
341-379	1392106	DKDPNFKDQVILLNKHIDAYKTFPPTEPKKDKKKKADET	D377Y	B.1.617.2	phage display	[5]
358-381	1311663	DAYKTFPPTEPKKDKKKKADETQA	D377Y	B.1.617.2	microarray	[13]
361-378	1087597	KTFPPTEPKKDKKKKADE	D377Y, R385K	B.1.617.2	ELISA	[14]
361-380	1392251	KTFPPTEPKKDKKKKADETQ	D377Y	B.1.617.2	ELISA	[4]
362-377	1697880	TFPPTEPKKDKKKKAD	D377Y	B.1.617.2	microarray	[3]
363-378	1658346	FPPTEPKKDKKKKADE	D377Y	B.1.617.2	microarray	[3]
363-408	1311678	FPPTEPKKDKKKKADETQALPQRQKKQQTVTLLPAADLDDFSKQLQ	D377Y, R385K	B.1.617.2	microarray	[13]
364-378	1542003	PPTEPKKDKKKKADE	D377Y	B.1.617.2	ELISA, microarray	[1,2]
364-379	1686290	PPTEPKKDKKKKADET	D377Y	B.1.617.2	microarray	[3]
364-385	1870805	PPTEPKKDKKKKADETQALPQR	D377Y, R385K	B.1.617.2	ELISA	[11]
365-391	1919836	PTEPKKDKKKKADETQALPQRQKKQQT	D377Y, R385K	B.1.617.2	ELISA	[10]
366-380	1310210	TEPKKDKKKKADETQ	D377Y	B.1.617.2	microarray	[2]
366-395	1862494	TEPKKDKKKKADETQALPQRQKKQQTVTLL	D377Y, R385K	B.1.617.2	ELISA	[15]
367-381	2001035	EPKKDKKKKADETQA	D377Y	B.1.617.2	microarray	[1]
367-382	1655029	EPKKDKKKKADETQAL	D377Y	B.1.617.2	microarray	[3]
368-382	1541962	PKKDKKKKADETQAL	D377Y	B.1.617.2	ELISA	[7]
368-383	1685675	PKKDKKKKADETQALP	D377Y	B.1.617.2	microarray	[3]
369-380	1459304	KKDKKKKADETQ	D377Y	B.1.617.2	microarray	[6]
369-384	1670649	KKDKKKKADETQALPQ	D377Y	B.1.617.2	microarray	[3]
369-386	1087574	KKDKKKKADETQALPQRQ	D377Y, R385K	B.1.617.2	ELISA	[14]
370-384	1540947	KDKKKKADETQALPQ	D377Y	B.1.617.2	ELISA, microarray	[1,7]
370-385	1457322	KDKKKKADETQALPQR	D377Y, R385K	B.1.617.2	microarray	[3,6]
371-385	1310080	DKKKKADETQALPQR	D377Y, R385K	B.1.617.2	microarray	[2]
371-386	1651126	DKKKKADETQALPQRQ	D377Y, R385K	B.1.617.2	microarray	[3]
371-390	1392108	DKKKKADETQALPQRQKKQQ	D377Y, R385K	B.1.617.2	ELISA	[4]
372-387	1670696	KKKKADETQALPQRQK	D377Y, R385K	B.1.617.2	microarray	[3]
373-387	1312850	KKKADETQALPQRQK	D377Y, R385K	B.1.617.2	microarray	[1,8]
373-388	1670695	KKKADETQALPQRQKK	D377Y, R385K	B.1.617.2	microarray	[3]
374-388	1541021	KKADETQALPQRQKK	D377Y, R385K	B.1.617.2	ELISA	[7]
374-389	1670626	KKADETQALPQRQKKQ	D377Y, R385K	B.1.617.2	microarray	[3]
375-390	1669082	KADETQALPQRQKKQQ	D377Y, R385K	B.1.617.2	microarray	[3]
376-390	1310054	ADETQALPQRQKKQQ	D377Y, R385K	B.1.617.2	microarray	[1]
376-391	1377343	ADETQALPQRQKKQQT	D377Y, R385K	B.1.617.2	microarray	[3]
376-397	1377344	ADETQALPQRQKKQQTVTLLPA	D377Y, R385K	B.1.617.2	microarray	[3]

376-400	1407393	ADETQALPQRQKKQQTVTLLP AADL	D377Y, R385K	B.1.617.2	microarray	[6]
377-387	1339955	DETQALPQRQK	D377Y, R385K	B.1.617.2	microarray	[8]
377-388	1420342	DETQALPQRQKK	D377Y, R385K	B.1.617.2	immunoprecipitation, microarray	[6,16]
377-391	1312288	DETQALPQRQKKQQT	D377Y, R385K	B.1.617.2	microarray	[8]
377-392	1378502	DETQALPQRQKKQQTV	D377Y, R385K	B.1.617.2	microarray	[16]
378-393	1380089	ETQALPQRQKKQQTVT	R385K	B.1.617.2	microarray	[3]
379-393	2001340	TQALPQRQKKQQTVT	R385K	B.1.617.2	microarray	[1]
379-394	1389884	TQALPQRQKKQQTVTL	R385K	B.1.617.2	microarray	[3]
379-419	1862941	TQALPQRQKKQQTVTLLPAAD LDDFSKQLQQSMSSADSTQA	R385K	B.1.617.2	ELISA, immunostaining	[17]
380-395	1386999	QALPQRQKKQQTVTLL	R385K	B.1.617.2	microarray	[3]
381-395	1310061	ALPQRQKKQQTVTLL	R385K	B.1.617.2	microarray	[2,18]
381-396	1377653	ALPQRQKKQQTVTLLP	R385K	B.1.617.2	microarray	[3]
381-419	1334453	ALPQRQKKQQTVTLLPAADLD DFSKQLQQSMSSADSTQA	R385K	B.1.617.2	phage display, western blot	[5,19]
382-397	1384883	LPQRQKKQQTVTLLPA	R385K	B.1.617.2	microarray, western blot	[3,20]
382-396	1541442	LPQRQKKQQTVTLLP	R385K	B.1.617.2	ELISA, microarray	[1,7]
382-405	1311750	LPQRQKKQQTVTLLPAADLDD FSK	R385K	B.1.617.2	microarray	[13]
383-398	1686384	PQRQKKQQTVTLLPAA	R385K	B.1.617.2	microarray	[3]
384-398	1542180	QRQKKQQTVTLLPAA	R385K	B.1.617.2	ELISA	[7]
384-399	1387379	QRQKKQQTVTLLPAAD	R385K	B.1.617.2	microarray	[3]
384-416	1387381	QRQKKQQTVTLLPAADLDDFS KQLQQSMSSADS	R385K	B.1.617.2	microarray	[3]
385-396	1499582	RQKKQQTVTLLP	R385K	B.1.617.2	immunoprecipitation	[16]
385-399	1313484	RQKKQQTVTLLPAAD	R385K	B.1.617.2	microarray	[1,8]
385-400	1387865	RQKKQQTVTLLPAADL	R385K	B.1.617.2	microarray	[3]

Table S3. List of variable T-cell epitopes of N protein of SARS-CoV-2 with confirmed binding to HLA molecules.

Position	IEDB ID	Epitope sequence	Mutation	Strain	Allele	Method	Reference
1-15	1313173	MSDNGPQNQRNAPRI	P13S, P13L	B.1.351, B.1.1.529	HLA class II	ICS IFNγ release	[21]
1-17	1309131	MSDNGPQNQRNAPRITF	P13S, P13L	B.1.351, B.1.1.529	HLA-B*27:05, HLA-B*27:06	ELISPOT IFNγ release	[22]
5-17	1317715	GPQNQRNAPRITF	P13S, P13L	B.1.351, B.1.1.529	HLA-B*07:02	activation	[23]

6-20	1323019	PQNQRNAPRITFGGP	P13S, P13L	B.1.351, B.1.1.529	HLA class II	ICS IFNγ release, activation	[21,23]
8-25	1309133	NQRNAPRITFGGPSDSTG	P13S, P13L	B.1.351, B.1.1.529	HLA-B*27:05, HLA-B*27:06	ELISPOT IFNγ release	[22]
9-17	1309136	QRNAPRITF	P13S, P13L	B.1.351, B.1.1.529	HLA-B*27:05, HLA-C*07:01	ELISPOT IFNγ release, qualitative binding, activation	[24–26]
9-27	1494865	QRNAPRITFGGPSDSTGSN	P13S, P13L	B.1.351, B.1.1.529	HLA class II	ELISA IFNγ release	[27]
11-25	1322297	NAPRITFGGPSDSTG	P13S, P13L	B.1.351, B.1.1.529	HLA-DQB1*03:01	ICS IFNγ release, activation	[23]
12-20	1330992	APRITFGGP	P13S, P13L	B.1.351, B.1.1.529	HLA-A*02:01, HLA-B*07:02	qualitative binding	[28]
17-31	1312422	FGGPSDSTGSNQNGE	E31 del	B.1.1.529	HLA class II	ELISPOT IFNγ release	[29]
21-35	1313538	SDSTGSNQNGERSGA	ERS31-33 del	B.1.1.529	HLA class II	ICS IFNγ release	[21]
26-40	1542535	SNQNGERSGARSKQR	ERS31-33 del	B.1.1.529	HLA class II	ICS IFNγ release	[21]
31-45	1397276	ERSGARSKQRRPQGL	ERS31-33 del	B.1.1.529	HLA class II	ICS IFNγ release	[21]
45-63	1470968	LPNNTASWFTALTQHGKED	D63G	B.1.617.2	HLA class II	ELISA IFNγ release	[27]
49-68	1869725	TASWFTALTQHGKEDLKFPR	D63G	B.1.617.2	HLA-DRB1*04:01, HLA-DRB5*01:01	qualitative binding	[27]
50-64	1310292	ASWFTALTQHGKEDL	D63G	B.1.617.2	HLA-DRB4*01:03	ELISPOT IFNγ release	[30,31]
51-65	1172352	SWFTALTQHGKEDLK	D63G	B.1.617.2	HLA-DRB	ICS IFNγ release, activation	[21,23]
56-70	1321406	LTQHGKEDLKFPRGQ	D63G	B.1.617.2	HLA class II	activation	[23]
63-75	1312809	KEDLKFPRGQGVPIN	D63G	B.1.617.2	HLA class II	ICS IFNγ release	[21]
66-80	1316855	FPRGQGVPINTNSSP	P80R	P.1	HLA class II	ICS IFNγ release, activation	[21,23]
71-85	1317881	GVPINTNSSPDDQIG	P80R	P.1	HLA class II	ICS IFNγ release, activation	[21,23]
75-87	1075010	NTNSSPDDQIGYY	P80R	P.1	HLA-A*01:01	qualitative binding, activation	[23,32]
76-90	1542862	TNSSPDDQIGYYRRA	P80R	P.1	HLA class II	ICS IFNγ release	[21]
77-87	1541833	NSSPDDQIGYY	P80R	P.1	HLA-A*01:01	qualitative binding	[33]
78-87	1542585	SSPDDQIGYY	P80R	P.1	HLA class I, HLA-B*35:02, HLA-B*35:01, HLA-C*04:01	ELISA IFNγ release	[30]
78-88	1333792	SSPDDQIGYYR	P80R	P.1	HLA-A*01:01	qualitative binding	[25]
79-87	1310816	SPDDQIGYY	D63G	B.1.617.2	HLA-B*35:01	activation	[23]
191-205	1597796	RNSSRNSTPGSSKRT	R203K G204R T205I	B.1.351, P.1, B.1.617.2, B.1.1.529	HLA class II	ICS IFNγ release	[21]

196-210	1597775	NSTPGSSKRTSPARM	R203K G204R T205I	B.1.351, P.1, B.1.617.2, B.1.1.529	HLA class II	ICS IFNγ release	[21]
201-209	1075062	SSRGTSPAR	R203K G204R T205I	B.1.351, P.1, B.1.617.2, B.1.1.529	HLA class I	activation	[34]
201-215	1597811	SSKRTSPARMAGNGG	R203K G204R T205I G215C	B.1.351, P.1, B.1.617.2, B.1.1.529	HLA class II	ICS IFNγ release	[21]
206-220	1542543	SPARMAGNGGDAALA	G215C	B.1.617.2	HLA class II	ICS IFNγ release	[21]
209-223	1313463	RMAGNGGDAALALLL	G215C	B.1.617.2	HLA class II	ELISA IFNγ release	[27]
209-227	1498997	RMAGNGGDAALALLLD RL	G215C	B.1.617.2	HLA class II	ELISA IFNγ release	[27]
211-225	1314288	AGNGGDAALALLLLD	G215C	B.1.617.2	HLA-DRB	ICS IFNγ release, activation	[27]
215-223	2134089	GDAALALLL	G215C	B.1.617.2	HLA-A*02:01	qualitative binding	[35]
366-379	1310210	TEPKKDKKKKADETQ	D377Y	B.1.617.2	HLA class II	ICS IFNγ release	[21]
371-385	1310080	DKKKKADETQALPQR	D377Y, R385K	B.1.617.2	HLA class II	ICS IFNγ release	[21]
376-390	1310054	ADETQALPQRQKKQQ	D377Y, R385K	B.1.617.2	HLA class II	ICS IFNγ release	[21]
381-395	1310061	ALPQRQKKQQTVTLL	R385K	B.1.617.2	HLA class II	ICS IFNγ release	[21]
385-404	1868935	RQKKQQTVTLLPAADLDD FS	R385K	B.1.617.2	HLA-DRB1*01:01	qualitative binding	[36]

References

1. Hotop, S.-K.; Reimering, S.; Shekhar, A.; Asgari, E.; Beutling, U.; Dahlke, C.; Fathi, A.; Khan, F.; Lütgehetmann, M.; Ballmann, R.; et al. Peptide Microarrays Coupled to Machine Learning Reveal Individual Epitopes from Human Antibody Responses with Neutralizing Capabilities against SARS-CoV-2. *Emerging Microbes & Infections* **2022**, *11*, 1037–1048, doi:10.1080/22221751.2022.2057874.
2. Voss, C.; Esmail, S.; Liu, X.; Knauer, M.J.; Ackloo, S.; Kaneko, T.; Lowes, L.; Stogios, P.; Seitova, A.; Hutchinson, A.; et al. Epitope-Specific Antibody Responses Differentiate COVID-19 Outcomes and Variants of Concern. *JCI Insight* **2021**, *6*, e148855, doi:10.1172/jci.insight.148855.
3. Heffron, A.S.; McIlwain, S.J.; Amjadi, M.F.; Baker, D.A.; Khullar, S.; Sethi, A.K.; Palmenberg, A.C.; Shelef, M.A.; O'Connor, D.H.; Ong, I.M. *The Landscape of Antibody Binding in SARS-CoV-2 Infection*; Immunology, 2020;
4. Gregory, D.J.; Vannier, A.; Duey, A.H.; Roady, T.J.; Dzeng, R.K.; Pavlovic, M.N.; Chapin, M.H.; Mukherjee, S.; Wilmot, H.; Chronos, N.; et al. Repertoires of SARS-CoV-2 Epitopes Targeted by Antibodies Vary According to Severity of COVID-19. *Virulence* **2022**, *13*, 890–902, doi:10.1080/21505594.2022.2073025.
5. Stoddard, C.I.; Galloway, J.; Chu, H.Y.; Shipley, M.M.; Sung, K.; Itell, H.L.; Wolf, C.R.; Logue, J.K.; Magedson, A.; Garrett, M.E.; et al. Epitope Profiling Reveals Binding Signatures of SARS-CoV-2 Immune Response in Natural Infection and Cross-Reactivity with Endemic Human CoVs. *Cell Reports* **2021**, *35*, 109164, doi:10.1016/j.celrep.2021.109164.
6. Mishra, N.; Huang, X.; Joshi, S.; Guo, C.; Ng, J.; Thakkar, R.; Wu, Y.; Dong, X.; Li, Q.; Pinapati, R.S.; et al. Immunoreactive Peptide Maps of SARS-CoV-2. *Commun Biol* **2021**, *4*, 225, doi:10.1038/s42003-021-01743-9.
7. Schwarz, T.; Heiss, K.; Mahendran, Y.; Casilag, F.; Kurth, F.; Sander, L.E.; Wendtner, C.-M.; Hoechstetter, M.A.; Müller, M.A.; Sekul, R.; et al. SARS-CoV-2 Proteome-Wide Analysis Revealed Significant Epitope Signatures in COVID-19 Patients. *Front. Immunol.* **2021**, *12*, 629185, doi:10.3389/fimmu.2021.629185.

8. Holenya, P.; Lange, P.J.; Reimer, U.; Woltersdorf, W.; Panterodt, T.; Glas, M.; Wasner, M.; Eckey, M.; Drosch, M.; Hollidt, J.; et al. Peptide Microarray-based Analysis of Antibody Responses to SARS-CoV-2 Identifies Unique Epitopes with Potential for Diagnostic Test Development. *Eur. J. Immunol.* **2021**, *51*, 1839–1849, doi:10.1002/eji.202049101.
9. Yu, J.; Qin, Z.; Liu, X.; He, X.; Yao, J.; Zhou, X.; Wen, K.; Yu, N.; Wu, Q.; Xiao, W.; et al. High-Specificity Targets in SARS-CoV-2 N Protein for Serological Detection and Distinction from SARS-CoV. *Computers in Biology and Medicine* **2022**, *143*, 105272, doi:10.1016/j.compbiomed.2022.105272.
10. Kumar, G.; Sterrett, S.; Hall, L.; Tabengwa, E.; Honjo, K.; Larimer, M.; Davis, R.S.; Goepfert, P.A.; Larimer, B.M. Comprehensive Mapping of SARS-CoV-2 Peptide Epitopes for Development of a Highly Sensitive Serological Test for Total and Neutralizing Antibodies. *Protein Engineering, Design and Selection* **2022**, *35*, gzab033, doi:10.1093/protein/gzab033.
11. Garanina, E.; Hamza, S.; Stott-Marshall, R.J.; Martynova, E.; Markelova, M.; Davidyuk, Y.; Shakirova, V.; Kaushal, N.; Baranwal, M.; Khaertynova, I.M.; et al. Antibody and T Cell Immune Responses to SARS-CoV-2 Peptides in COVID-19 Convalescent Patients. *Front. Microbiol.* **2022**, *13*, 842232, doi:10.3389/fmicb.2022.842232.
12. Lu, S.; Xie, X.; Zhao, L.; Wang, B.; Zhu, J.; Yang, T.; Yang, G.; Ji, M.; Lv, C.; Xue, J.; et al. The Immunodominant and Neutralization Linear Epitopes for SARS-CoV-2. *Cell Reports* **2021**, *34*, 108666, doi:10.1016/j.celrep.2020.108666.
13. Shrock, E.; Fujimura, E.; Kula, T.; Timms, R.T.; Lee, I.-H.; Leng, Y.; Robinson, M.L.; Sie, B.M.; Li, M.Z.; Chen, Y.; et al. Viral Epitope Profiling of COVID-19 Patients Reveals Cross-Reactivity and Correlates of Severity. *Science* **2020**, *370*, eabd4250, doi:10.1126/science.abd4250.
14. Amrun, S.N.; Lee, C.Y.-P.; Lee, B.; Fong, S.-W.; Young, B.E.; Chee, R.S.-L.; Yeo, N.K.-W.; Torres-Ruesta, A.; Carissimo, G.; Poh, C.M.; et al. Linear B-Cell Epitopes in the Spike and Nucleocapsid Proteins as Markers of SARS-CoV-2 Exposure and Disease Severity. *EBioMedicine* **2020**, *58*, 102911, doi:10.1016/j.ebiom.2020.102911.
15. Herrscher, C.; Eymieux, S.; Gaborit, C.; Blasco, H.; Marlet, J.; Stefic, K.; Roingeard, P.; Grammatico-Guillon, L.; Hourioux, C. ELISA-Based Analysis Reveals an Anti-SARS-CoV-2 Protein Immune Response Profile Associated with Disease Severity. *JCM* **2022**, *11*, 405, doi:10.3390/jcm11020405.
16. Haynes, W.A.; Kamath, K.; Bozekowski, J.; Baum-Jones, E.; Campbell, M.; Casanovas-Massana, A.; Daugherty, P.S.; Dela Cruz, C.S.; Dhal, A.; Farhadian, S.F.; et al. High-Resolution Epitope Mapping and Characterization of SARS-CoV-2 Antibodies in Large Cohorts of Subjects with COVID-19. *Commun Biol* **2021**, *4*, 1317, doi:10.1038/s42003-021-02835-2.
17. Tian, Y.; Zhang, G.; Liu, H.; Ding, P.; Jia, R.; Zhou, J.; Chen, Y.; Qi, Y.; Du, J.; Liang, C.; et al. Screening and Identification of B Cell Epitope of the Nucleocapsid Protein in SARS-CoV-2 Using the Monoclonal Antibodies. *Appl Microbiol Biotechnol* **2022**, *106*, 1151–1164, doi:10.1007/s00253-022-11769-6.
18. Yang, L.; Liang, T.; Pierson, L.M.; Wang, H.; Fletcher, J.K.; Wang, S.; Bao, D.; Zhang, L.; Huang, Z.; Zheng, W.; et al. SARS-CoV-2 Epitopes Following Infection and Vaccination Overlap Known Neutralizing Antibody Sites. *Research* **2022**, *2022*, 1–14, doi:10.34133/2022/9769803.
19. Terry, J.S.; Anderson, L.BR.; Scherman, M.S.; McAlister, C.E.; Perera, R.; Schountz, T.; Geiss, B.J. Development of a SARS-CoV-2 Nucleocapsid Specific Monoclonal Antibody. *Virology* **2021**, *558*, 28–37, doi:10.1016/j.virol.2021.01.003.
20. Yamaoka, Y.; Miyakawa, K.; Jeremiah, S.S.; Funabashi, R.; Okudela, K.; Kikuchi, S.; Katada, J.; Wada, A.; Takei, T.; Nishi, M.; et al. Highly Specific Monoclonal Antibodies and Epitope Identification against SARS-CoV-2 Nucleocapsid Protein for Antigen Detection Tests. *Cell Reports Medicine* **2021**, *2*, 100311, doi:10.1016/j.xcrm.2021.100311.
21. Heide, J.; Schulte, S.; Kohsar, M.; Brehm, T.T.; Herrmann, M.; Karsten, H.; Marget, M.; Peine, S.; Johansson, A.M.; Sette, A.; et al. Broadly Directed SARS-CoV-2-Specific CD4+ T Cell Response Includes Frequently Detected Peptide Specificities within the Membrane and Nucleoprotein in Patients with Acute and Resolved COVID-19. *PLoS Pathog* **2021**, *17*, e1009842, doi:10.1371/journal.ppat.1009842.
22. Peng, Y.; Mentzer, A.J.; Liu, G.; Yao, X.; Yin, Z.; Dong, D.; Dejnirattisai, W.; Rostron, T.; Supasa, P.; Liu, C.; et al. Broad and Strong Memory CD4+ and CD8+ T Cells Induced by SARS-CoV-2 in UK Convalescent Individuals Following COVID-19. *Nat Immunol* **2020**, *21*, 1336–1345, doi:10.1038/s41590-020-0782-6.

23. Tarke, A.; Sidney, J.; Kidd, C.K.; Dan, J.M.; Ramirez, S.I.; Yu, E.D.; Mateus, J.; da Silva Antunes, R.; Moore, E.; Rubiro, P.; et al. Comprehensive Analysis of T Cell Immunodominance and Immunoprevalence of SARS-CoV-2 Epitopes in COVID-19 Cases. *Cell Reports Medicine* **2021**, *2*, 100204, doi:10.1016/j.xcrm.2021.100204.
24. van den Dijssel, J.; Hagen, R.R.; de Jongh, R.; Steenhuis, M.; Rispens, T.; Geerdes, D.M.; Mok, J.Y.; Kragten, A.H.; Duurland, M.C.; Verstegen, N.J.; et al. Parallel Detection of SARS-CoV -2 Epitopes Reveals Dynamic Immunodominance Profiles of CD8 $^{+}$ T Memory Cells in Convalescent COVID -19 Donors. *Clin & Trans Imm* **2022**, *11*, doi:10.1002/cti2.1423.
25. Saini, S.K.; Hersby, D.S.; Tamhane, T.; Povlsen, H.R.; Hernandez, S.P.A.; Nielsen, M.; Gang, A.O.; Hadrup, S.R. SARS-CoV-2 Genome-Wide T Cell Epitope Mapping Reveals Immunodominance and Substantial CD8 $^{+}$ T Cell Activation in COVID-19 Patients. *Sci. Immunol.* **2021**, *6*, eabf7550, doi:10.1126/sciimmunol.abf7550.
26. de Silva, T.I.; Liu, G.; Lindsey, B.B.; Dong, D.; Moore, S.C.; Hsu, N.S.; Shah, D.; Wellington, D.; Mentzer, A.J.; Angyal, A.; et al. The Impact of Viral Mutations on Recognition by SARS-CoV-2 Specific T Cells. *iScience* **2021**, *24*, 103353, doi:10.1016/j.isci.2021.103353.
27. Verhagen, J.; van der Meijden, E.D.; Lang, V.; Kremer, A.E.; Völkl, S.; Mackensen, A.; Aigner, M.; Kremer, A.N. Human CD4+ T Cells Specific for Dominant Epitopes of SARS-CoV-2 Spike and Nucleocapsid Proteins with Therapeutic Potential. *Clinical and Experimental Immunology* **2021**, *205*, 363–378, doi:10.1111/cei.13627.
28. Weingarten-Gabbay, S.; Klaeger, S.; Sarkizova, S.; Pearlman, L.R.; Chen, D.-Y.; Gallagher, K.M.E.; Bauer, M.R.; Taylor, H.B.; Dunn, W.A.; Tarr, C.; et al. Profiling SARS-CoV-2 HLA-I Peptidome Reveals T Cell Epitopes from out-of-Frame ORFs. *Cell* **2021**, *184*, 3962-3980.e17, doi:10.1016/j.cell.2021.05.046.
29. Lin, J.; Law, R.; Korosec, C.S.; Zhou, C.; Koh, W.H.; Ghaemi, M.S.; Samaan, P.; Ooi, H.K.; Matveev, V.; Yue, F.; et al. Longitudinal Assessment of SARS-CoV-2-Specific T Cell Cytokine-Producing Responses for 1 Year Reveals Persistence of Multicytokine Proliferative Responses, with Greater Immunity Associated with Disease Severity. *J Virol* **2022**, *96*, e00509-22, doi:10.1128/jvi.00509-22.
30. Titov, A.; Shaykhutdinova, R.; Shcherbakova, O.V.; Serdyuk, Y.V.; Sheetikov, S.A.; Zornikova, K.V.; Maleeva, A.V.; Khmelevskaya, A.; Dianov, D.V.; Shakirova, N.T.; et al. Immunogenic Epitope Panel for Accurate Detection of Non-Cross-Reactive T Cell Response to SARS-CoV-2. *JCI Insight* **2022**, *7*, e157699, doi:10.1172/jci.insight.157699.
31. Heitmann, J.S.; Bilich, T.; Tandler, C.; Nelde, A.; Maringer, Y.; Marconato, M.; Reusch, J.; Jäger, S.; Denk, M.; Richter, M.; et al. A COVID-19 Peptide Vaccine for the Induction of SARS-CoV-2 T Cell Immunity. *Nature* **2022**, *601*, 617–622, doi:10.1038/s41586-021-04232-5.
32. Minervina, A.A.; Pogorelyy, M.V.; Kirk, A.M.; Crawford, J.C.; Allen, E.K.; Chou, C.-H.; Mettelman, R.C.; Allison, K.J.; Lin, C.-Y.; Brice, D.C.; et al. SARS-CoV-2 Antigen Exposure History Shapes Phenotypes and Specificity of Memory CD8+ T Cells. *Nat Immunol* **2022**, *23*, 781–790, doi:10.1038/s41590-022-01184-4.
33. Nagler, A.; Kalaora, S.; Barbolin, C.; Gangaev, A.; Ketelaars, S.L.C.; Alon, M.; Pai, J.; Benedek, G.; Yahalom-Ronen, Y.; Erez, N.; et al. Identification of Presented SARS-CoV-2 HLA Class I and HLA Class II Peptides Using HLA Peptidomics. *Cell Reports* **2021**, *35*, 109305, doi:10.1016/j.celrep.2021.109305.
34. Snyder, T.M.; Gittelman, R.M.; Klinger, M.; May, D.H.; Osborne, E.J.; Taniguchi, R.; Zahid, H.J.; Kaplan, I.M.; Dines, J.N.; Noakes, M.T.; et al. *Magnitude and Dynamics of the T-Cell Response to SARS-CoV-2 Infection at Both Individual and Population Levels*; Infectious Diseases (except HIV/AIDS), 2020;
35. Jm, F.; D, L.-E.; A, D.; C, T.; J, L.; C, D.; A, H.; V, R.; G, L.; Y, W.; et al. Allelic Variation in Class I HLA Determines CD8+ T Cell Repertoire Shape and Cross-Reactive Memory Responses to SARS-CoV-2. *Science immunology* **2022**, *7*, doi:10.1126/sciimmunol.abk3070.
36. Johansson, A.M.; Malhotra, U.; Kim, Y.G.; Gomez, R.; Krist, M.P.; Wald, A.; Koelle, D.M.; Kwok, W.W. Cross-Reactive and Mono-Reactive SARS-CoV-2 CD4+ T Cells in Prepandemic and COVID-19 Convalescent Individuals. *PLoS Pathog* **2021**, *17*, e1010203, doi:10.1371/journal.ppat.1010203.
